# Neural Determinants of Task Performance during Feature-Based Attention in Human Cortex

**DOI:** 10.1523/ENEURO.0375-17.2018

**Published:** 2018-02-28

**Authors:** Michael Jigo, Mengyuan Gong, Taosheng Liu

**Affiliations:** 1 Department of Psychology, Michigan State University, East Lansing, MI 48824; 2 Center for Neural Science, New York University, New York, NY 10003

**Keywords:** covert attention, fMRI, frontoparietal cortex, multivariate analysis, TMS

## Abstract

Studies of feature-based attention have associated activity in a dorsal frontoparietal network with putative attentional priority signals. Yet, how this neural activity mediates attentional selection and whether it guides behavior are fundamental questions that require investigation. We reasoned that endogenous fluctuations in the quality of attentional priority should influence task performance. Human subjects detected a speed increment while viewing clockwise (CW) or counterclockwise (CCW) motion (baseline task) or while attending to either direction amid distracters (attention task). In an fMRI experiment, direction-specific neural pattern similarity between the baseline task and the attention task revealed a higher level of similarity for correct than incorrect trials in frontoparietal regions. Using transcranial magnetic stimulation (TMS), we disrupted posterior parietal cortex (PPC) and found a selective deficit in the attention task, but not in the baseline task, demonstrating the necessity of this cortical area during feature-based attention. These results reveal that frontoparietal areas maintain attentional priority that facilitates successful behavioral selection.

## Significance Statement

To cope with the computational limits of visual processing, the brain selectively prioritizes a subset of visual input. The selection of visual features, such as color and motion, has been associated with activity in a frontoparietal cortical network. Yet, the role this activity plays in mediating selection and influencing behavior is not clear. Using fMRI, we show that neural activity patterns in several frontoparietal areas correlated with task performance. Furthermore, neurodisruption of the posterior parietal cortex (PPC) using transcranial magnetic stimulation (TMS) selectively impaired feature selection. These results provide the first evidence that the neural representation of prioritized features in frontoparietal areas play a causal role in selecting visual features.

## Introduction

Visual attention allows us to select relevant information from the visual scene for prioritized processing. Given the profound effect of attention on perception (Simons and Rensink, 2005), it is important to understand how attention is controlled both at the behavioral and neural level (Corbetta and Shulman, 2002).

Theories of attention have postulated that attentional priority, which represents the behavioral importance of visual objects, guides selection during perceptual processing (Wolfe, 1994; Desimone and Duncan, 1995; Deco and Rolls, 2004). On the neural level, attentional priority signals have been linked to activity in dorsal parietal and frontal areas. In particular, priority for spatial locations is thought to be represented by spatially selective neural responses in these areas (Silver and Kastner, 2009; Bisley and Goldberg, 2010). This idea has been strongly supported by neurophysiological and neuroimaging studies (for review, see Buschman and Kastner, 2015). In addition to spatial locations, attention can also be directed to nonspatial properties such as visual features (Yantis, 2000), and recent work has begun to characterize priority signals for feature-based attention (Serences and Boynton, 2007; Liu et al., 2011; Liu and Hou, 2013; Ester et al., 2016). Specifically, these studies have found that patterns of neural activity in visual and dorsal frontoparietal areas, in particular, areas along the intraparietal sulcus and the precentral sulcus, can be used to decode the attended feature value (e.g., red vs green color, leftward vs rightward motion). Although these studies demonstrate that neural activity patterns vary with the attended feature, they do not reveal how this activity mediates attentional selection and whether it is causally involved in feature selection and task performance.

Here, we investigate the neural-behavioral relation in regions that have been implicated in the selection of visual features. We reasoned that if neural activity encodes attentional priority for visual features, then such activity should be related to behavioral performance in a task requiring feature-based selection. In an fMRI experiment, we instructed human subjects to attend to one of two superimposed motion directions and perform a speed detection at psychophysical threshold (attention task). If neural activity in this task encodes priority for features, the quality of such signals should be better for correct than incorrect trials. To provide a benchmark to evaluate the quality of the priority signal, we measured neural activity in a separate task where subjects viewed a single motion direction (baseline task). We hypothesized that if attentional selection in the attention task is successful, then neural activity will resemble that in the baseline task, inasmuch as attention reduces the influence from the distracter. Conversely, neural activity for unsuccessful selection in the attention task would share less resemblance to that in the baseline task. Thus, we predict that the neural pattern similarity between the attention and baseline task would vary with task performance.

To assess the causal role of neural activity on feature-based selection, we used transcranial magnetic stimulation (TMS) to disrupt identified neural signals for attentional priority. We further hypothesized that if these signals represent priority for features, then neurodisruption would impair performance in the attention task, which requires feature-based selection, whereas it would impair performance less (or not at all) in the baseline task, which does not require feature-based selection. Conversely, we hypothesized that if these signals are related to general motion processing, then neurodisruption would produce equivalent impairments in both the attention and baseline task. To test these predictions, we unilaterally targeted representative brain areas that were identified as potential loci for maintaining attentional priority or stimulus processing.

## Materials and Methods

### fMRI experiment

#### Subjects

Twelve subjects participated in the imaging experiment (five males, 20–29 years old). All subjects were neurologically intact, had normal or corrected-to-normal vision, and were recruited from the Michigan State University community (undergraduate and graduate students). They gave written informed consent under the study protocol approved by the Institutional Review Board at Michigan State University and were remunerated at a rate of $20 per hour.

#### Visual display and stimuli

Visual stimuli were generated using MGL (http://gru.stanford.edu/doku.php/mgl/overview), a set of OpenGL libraries running in Matlab (Mathworks). In the psychophysics laboratory, stimuli were presented on a 19” CRT monitor (resolution: 1024 × 768, 60-Hz refresh rate) and subjects had their heads stabilized by a chin rest that was positioned 85 cm away from the monitor. During MR scanning sessions, a DLP projector (Psychology Software Tools) projected the stimuli onto a rear-projection screen located in the scanner bore. Subjects viewed the screen via an angled mirror attached to the head coil at a viewing distance of 60 cm. The projector had a resolution of 1024 × 768 and was updated at 60 Hz.

The stimuli were composed of one or two dot fields that rotated in the clockwise (CW) or counterclockwise (CCW) direction with 60% motion coherence. Each dot field was contained within an annulus (eccentricity from 2.5° to 8°) that was centered on a central cross (size: 0.5°) and displayed on a black background. Each dot within a field (dot color: gray; dot size: 0.1°; density: 1.1 dots/deg^2^) had a lifetime of six frames (0.1 s) to deter subjects from tracking individual dots. During training and imaging sessions, the central cross was either yellow or cyan to help subjects remember the current response mapping (see *Tasks*).

#### Tasks

##### Attention task

**Figure 1.**
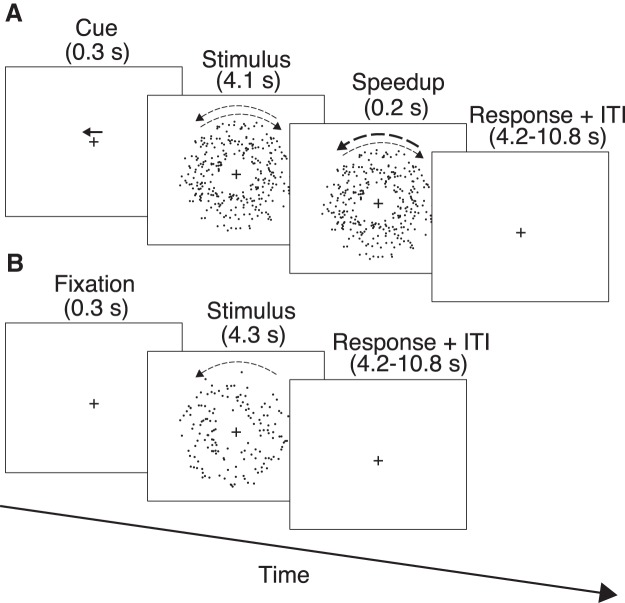
Schematic of the attention and baseline tasks. ***A***, Sequence of a valid trial in the attention task. Size of curved arrows illustrates the speed of rotation. The fixation cross is either yellow or cyan (color not shown). ***B***, Sequence of a target-absent trial in the baseline task. For ease of illustration, frames depict black stimuli on a white background (colors are reversed in the actual experiment).

At trial onset, subjects were cued to attend to CW or CCW motion by a rightward or leftward-pointing arrow cue, respectively (Fig. 1*A*). The cue appeared 0.77° above the central cross and persisted on the display for 0.3 s. The cue was then replaced with spatially overlapping CW and CCW dot fields that were displayed for 4.1 s. Each dot field rotated around the center of the annulus at a speed of 45°/s before a brief (0.2 s) speed increment occurred in either direction. Subjects were instructed to maintain fixation on the central cross and report whether the speedup occurred in the cued direction. Thus, task performance was contingent on the attended direction. If CW (CCW) was cued when a CW (CCW)-speedup occurred, and the subject reported the speedup, the trial was classified as a hit. Alternatively, if the subject failed to report the speedup, the trial was classified as a miss.

On 80% of trials, the speedup occurred in the cued direction (valid trials) and its magnitude was adjusted via best Parameter Estimation by Sequential Testing (PEST), an adaptive staircase procedure as implemented in the Palamedes toolbox (Prins and Kingdon, 2009), to maintain a hit rate (performance) of 65%. The best PEST procedure computed the maximum-likelihood estimate of an observer's speed increment threshold on each trial, on the basis of all previous responses. Performance was assumed to have the form of a Weibull function, with the slope fixed at 2, and the lapse rate and guess rate fixed at 1%. On invalid trials (20% of trials), a speedup occurred in the uncued direction (invalid trials) using the magnitude of the preceding valid trial (i.e., the speedup on invalid trials was not controlled via staircase).

For the attention task, we aimed to maximize the number of hit and miss trials to conduct our multivariate analyses. To accomplish this, we used a 4:1 validity ratio and titrated performance to an intermediate hit rate (65%). Although a lower hit rate, such as 50%, would mathematically maximize the number of hits and misses, such a low performance level could discourage subjects from using the cue.

An intertrial interval (ITI) followed the speedup, the ITI was 4.2 s on 40% of trials, 6.4 s on 30% of trials, 8.6 s on 20% of trials, and 10.8 s on 10% of trials. During this interval, subjects reported the presence or absence of a speedup in the cued direction via a “present” or “absent” response that was mapped onto a particular finger. To differentiate the observed neural response for target detection from that of a motor plan, we trained subjects to use two inverse response mappings that were indicated by the color of the central cross. When the cross was cyan, subjects made present and absent responses with their index and middle finger, respectively. When the cross was yellow, the mapping was reversed. The response mappings alternated across runs and were counterbalanced within each subject.

##### Baseline task

The baseline task (Fig. 1*B*) was identical to the attention task, with the following exceptions: (1) an arrow cue was not presented, hence, subjects viewed the central cross for 0.3 s at the beginning of each trial; (2) only one dot field was displayed; (3) a speedup occurred on 70% of trials (target-present trials) and on the other 30% of trials (target-absent trials), no speedup occurred; and (4) the hit rate was maintained at 50% via best PEST. These behavioral manipulations were used to maximize the number of correct rejections (when subjects reported absent on target-absent trials), as these served as the baseline neural patterns in our multivariate analyses. In particular, the low hit rate was chosen to dissuade subjects from making too many false positive reports, which would reduce the correct rejection rate.

#### Procedure

##### Training session

Before the imaging session, subjects completed at least two runs (50 trials/run) of each task in the psychophysics laboratory for practice. To ensure that subjects maintained fixation on the central cross, their left eye was recorded at a sampling rate of 1000 Hz using an Eyelink 1000 system (SR Research); the data were analyzed offline using custom Matlab scripts. Importantly, subjects were only allowed to proceed with the imaging session if their eye position was always within 2° of the central cross.

Because speedup events only occurred at the end of a trial, this could allow for a strategy where attention was only directed to the stimulus later in the trial. Thus, we also emphasized the importance of maintaining attention throughout each trial and all subjects reported compliance. We note that partial and/or inconsistent attentional engagement would likely reduce the attentional signal and increase noise in neural data. Hence, our observed effects might be even stronger if attention had been engaged more consistently throughout the stimulus duration.

##### Imaging session

Before functional images were collected, subjects completed, at most, 100 trials of each task while they lay in the scanner; these trials were used to calibrate the staircase to maintain the expected hit rate. Then, subjects completed 12 fMRI runs (30 trials/run), with six runs for each task in an alternating sequence. Each run began with an 8.8-s fixation period and lasted 338.8 s; the images collected during the fixation period were discarded to avoid magnetic saturation effects. For the attention task, cue direction (CW vs CCW) and validity (valid vs invalid) were randomly interleaved within each run; whereas, during the baseline task, motion direction (CW vs CCW) and trial type (target-present vs target-absent) were randomly interleaved within each run. Eye position was not monitored during this session.

#### MRI data acquisition

Imaging was performed on a GE Healthcare 3 T Sigma HDx MRI scanner, equipped with an eight-channel head coil, in the Department of Radiology at Michigan State University. For each subject, high-resolution anatomic images were acquired using a T1-weighted magnetization-prepared rapid-acquisition gradient echo sequence (field of view, 256 × 256 mm; 180 sagittal slices; 1-mm isotropic voxels). Functional images were acquired using a T2*-weighted echo planar imagining sequence (repetition time, 2.2 s; echo time, 30 ms; flip angle, 78°; matrix size, 64 × 64; in-plane resolution, 3 × 3 mm; slice thickness, 4 mm, interleaved, no gap). Thirty axial slices covering the whole brain were collected. In each scanning session, we also acquired a 2D T1-weighted anatomic image that had the same slice prescription as the functional scans but with higher in-plane resolution (0.75 × 0.75 × 4 mm). This image was used to align the functional data to the high-resolution anatomic images for each subject.

#### Retinotopic mapping

In an independent scanning session, we mapped each subject's occipital visual cortex and several parietal areas that contain topographic maps. For the occipital cortex, we used rotating wedges and expanding/contracting rings (eccentricity from 0.5° to 8.25°) to map the polar angle and radial component, respectively (Sereno et al., 1995; DeYoe et al., 1996; Engel et al., 1997). Four runs of the wedge stimuli and two runs of the ring stimuli were collected and averaged. A Fourier analysis was then applied to the averaged time course to derive the amplitude and phase of the response, the latter forming the polar angle map of the responses. Borders between areas were defined as phase reversals in the polar angle map of the visual field. The map was visualized on computationally flattened representations of the cortical surface, which were generated from a high-resolution anatomic image using FreeSurfer (http://surfer.nmr.mgh.harvard.edu) and custom Matlab code.

Parietal areas were mapped with a memory delayed saccade task that was modeled after previous studies on parietal topography (Sereno et al., 2001; Schluppeck et al., 2005; Konen and Kastner, 2008). Subjects fixated on a central point while a peripheral (∼10° radius) target dot was flashed for 500 ms. The flashed peripheral stimulus was quickly replaced by a ring of 100 distractor dots randomly positioned within a ring with a radius of 8.5–10.5°. The distractors remained on screen for 3 s, after which subjects made a saccade to the remembered position of the peripheral target and then immediately made a saccade back to the central fixation point. The position of the peripheral saccade target shifted around the periphery from trial to trial in either a CW or CCW direction, so that after eight trials the target completed one full cycle. A trial lasted 5 s and six cycles were completed in a single run. Two to four runs of the memory delayed saccade task were collected and averaged, then borders between parietal areas were defined as phase reversals in the polar angle map.

Finally, we presented moving versus stationary dots (eccentricity from 0.5° to 10°) in alternating blocks and localized the human motion-sensitive area as an area near the junction of the occipital and temporal cortex that responded more to moving than stationary dots (Watson et al., 1993). This area likely contained both MT and MST, so we refer to it as MT+.

Overall, we identified the following areas in each subject: V1, V2, V3, V3A/B, V4, MT+, V7/IPS0, IPS1, and IPS2. We did not observe a consistent boundary between V3A and V3B; hence, we defined an area that contained both and labeled it V3A/B. We adopted the definition of V4 as a hemifield representation anterior to V3v (Wandell et al., 2007). The V7/IPS0 nomenclature was adopted because its anatomic location is within the IPS in some hemispheres and shares a foveal representation with IPS1 (Swisher et al., 2007). We could not reliably observe borders for more anterior IPS regions such as IPS3 and IPS4 in all subjects.

#### fMRI data analysis

##### Preprocessing

Functional MRI data were analyzed using mrTools (http://gru.stanford.edu/doku.php/mrTools/overview) running in Matlab and custom code in Matlab. Preprocessing of functional data included head motion correction, linear detrending, and temporal high pass filtering at 0.01 Hz. The 2D T1-weighted image was used to compute the alignment between the functional images and the high-resolution T1-weighted image, using an automated robust image registration algorithm (Nestares and Heeger, 2000). Functional data were converted to percentage signal change by dividing the time course of each voxel by its mean signal over a run. Then, data for all runs of a task were concatenated, resulting in two time series. All region of interest (ROI) analyses were performed in individual subject's native anatomic space.

##### Univariate analysis: deconvolution

For the attention task, we fit each voxel’s time series with a general linear model containing five sets of regressors: four corresponding to the two directions during valid trials (CW vs CCW) crossed by response accuracy (hits vs misses) and the fifth corresponding to invalid trials. We note that due to the low proportion of invalid trials, false alarms were rather scarce (5 ± 5 false alarms across subjects), precluding a further separation into CW and CCW trials. In the analyses described below (see *Multivariate analysis*), we focus only on valid trials in the attention task; hence, hits and miss trials are referred to as correct and incorrect trials, respectively. For the baseline task, the general linear model contained seven sets of regressors: six corresponding to the two motion directions (CW vs CCW) crossed by detection type (hit vs miss vs correct rejection) and the seventh corresponding to false alarms. Each regressor modeled the fMRI response in a 17.6-s window after trial onset with a set of finite impulse responses. The design matrix was pseudo-inversed and multiplied by the time series to obtain an estimate of the hemodynamic response for each condition (deconvolution).

To obtain precise estimates of BOLD response amplitude for each subject, we averaged their deconvolved response across ROIs to obtain an overall response profile. These response profiles revealed a variable time-to-peak across subjects that ranged from 4.4 to 8.8 s after trial onset (time points 3–5). Voxel-wise estimates of response amplitude for each condition were then computed as the average deconvolved response from the time point immediately preceding the subject’s peak to the time point following the peak.

From the deconvolution model of each task, we obtained a goodness of fit measure (*r* ^2^ value) for each voxel, which was the amount of variance in the fMRI time series accounted for by the model (Gardner et al., 2005). The *r* ^2^ value indicated how much a voxel’s time series was driven by the task. For each subject, we used their *r* ^2^ map of the attention task to define two frontal ROIs. These ROIs were centered on voxels with maximal *r* ^2^ values that formed separate clusters along the precentral sulcus: one near the superior frontal sulcus and another near the inferior frontal sulcus. These clusters are not separate on the group map (Fig. 3*A*) but were distinct in individual subjects. At each location, we defined a ROI that extended to enclose the nearby sulcal junction while avoiding the precentral gyrus (motor cortex). The dorsal ROI coincided with the putative human frontal eye field (FEF) and we referred to the ventral ROI as the inferior frontal junction (IFJ); both areas were defined bilaterally.

##### Multivariate analysis: correlation

To measure attentional priority, we calculated the correlation between BOLD response patterns when subjects attended to a feature (attention task) and when they viewed the feature in isolation (baseline task; Fig. 4). The output of the correlation indexed the quality of the attentional priority signal, with low (high) correlations reflecting weak (strong) feature selection. Separate correlations were calculated for correct and incorrect trials.

BOLD response patterns were composed of voxel-wise response amplitudes that were estimated using the deconvolution analysis above, and represented the spatial pattern of neural activity evoked when each feature was viewed or attended. Attention patterns were constructed using the response to CW and CCW-cued motion, contingent on whether the behavioral response was correct or incorrect during the attention task (four patterns in total). Baseline patterns were constructed using the response to correct rejections for CW and CCW motion during the baseline task (two patterns in total). Because both the speedup (target) and its detection were absent during correct rejections, the associated neural response should have reflected feature processing, without the contribution from neural activities related to target detection. Correlations of attention and baseline patterns with matching features (e.g., attention CW and baseline CW) were calculated. The resulting correlation coefficients were averaged across features and statistical inferences were conducted on Fisher-transformed values. For each ROI, we conducted planned *t* tests between the feature selectivity on correct and incorrect trials.

##### Multivariate analysis: multivoxel pattern classification

For a separate measure of attentional priority, we trained a linear support vector machine (SVM; LIBSVM implementation; Chang and Lin, 2011) to discriminate between CW and CCW motion in the baseline task and then tested its ability to decode the motion directions when they were attended in the attention task, contingent on behavioral accuracy.

For this analysis, voxels were ranked by their *r* ^2^ value in the baseline task and the top 55 voxels in each ROI were used. Therefore, classification was based on the same number of voxels in each area. We note that our results were qualitatively identical when 35–145 voxels were used. We obtained single-trial BOLD responses (instances) for each voxel by averaging the time series between the time of peak response, as defined by a subject’s response profile, to the shortest possible trial duration (8.8 s; 5th time point). Due to the variable time-to-peak across subjects, the average window contained between one and three time points. Each instance was treated as a point in 55-dimensional space and was used to populate multivoxel responses (classes), contingent on trial type. CW and CCW Baseline classes were composed of BOLD response to correct rejection trials in the baseline task. CW and CCW Attention classes were composed of CW and CCW-cued trials in the attention task; separate classes were generated for correct and incorrect trials. Baseline classes were *z*-scored and used to train the SVM. Attention classes were *z*-scored using the mean and standard deviation of the training set, and used to test the SVM’s accuracy in predicting the attended feature. Correct and incorrect trials were tested separately. Classification accuracy for each ROI was assessed as the average across hemispheres. Planned *t* tests were conducted in each ROI to compare classification accuracy between correct and incorrect trials.

For ROIs that exhibited a difference in classification accuracy between correct and incorrect trials, we conducted a permutation analysis to assess whether classification accuracy was significantly above or below chance level. For each subject, SVMs were trained with Baseline classes and tested with random samples from Attention classes. Specifically, trial labels for all four Attention classes (accuracy crossed with motion direction) were shuffled and split into two classes of equal size. Then, classification accuracy was assessed for the shuffled data. At the individual subject level, this process was repeated 10,000 times to create a null distribution of classification accuracy for the ROI. Null distributions were averaged across hemispheres. To construct the group-level null distribution, a single value was randomly selected from each subject’s null distribution and the average value across subjects was calculated; this process was repeated 10,000 times to derive a null distribution for the ROI. Group-average classification accuracies below the 2.5th or above the 97.5th percentile were considered significantly below or above chance, respectively.

Note that the training and test data were based on different tasks in separate scanning runs and hence, entirely independent. Therefore, no leave-one-run-out cross-validation was necessary. On average, the training data contained 26 CW trials and 25 CCW trials. The test data contained 48 CW and 46 CCW correct trials, and 23 CW and 25 CCW incorrect trials.

##### Voxel exclusion criterion

Voxels with responses larger than 5% signal change were excluded from all analyses as they presumably reflected noise. At the group level, this criterion removed 4.1% of voxels across ROIs. We note that the exact exclusion criterion did not qualitatively impact our results.

#### Visualization of group data

All analyses were performed on individual subject data with predefined ROIs and all quantitative results reported were based on averages across individual subject results. We also performed group averaging of the individual maps to provide a visualization of the overall pattern of brain activity during the attention and baseline tasks. Each subject's two hemispherical surfaces were first imported into Caret and affine-transformed into the 711-2B space of the Washington University at St. Louis (Buckner et al., 2004). The surface was then inflated to a sphere and six landmarks were drawn, which were used for spherical registration to the landmarks in the Population-Average, Landmark- and Surface-based (PALS) atlas (Van Essen, 2005). Individual maps were transformed to the PALS atlas space and thresholded at a *r* ^2^ value of 0.12 in combination with a cluster constraint of 50 voxels. PALS-transformed maps were averaged across subjects and used solely for visualization purposes.

### TMS experiments

#### Subjects

In total, 27 subjects participated in the TMS experiments, with 12 participating in each experiment. Out of the total, one participated in all four experiments (author M.G.), four participated in three experiments (L/R parietal and MT+), eight participated in two experiments (L/R parietal: 3; R parietal and MT+: 3; one author, M.J.); L parietal and MT+: 1; MT+ and sham: 1), and 13 participated in one experiment (L parietal: two subjects; MT+: 1: sham: 10). All subjects were neurologically intact, had normal or corrected-to-normal vision, and were recruited from the Michigan State University community (undergraduate and graduate students). They gave written informed consent under the study protocol approved by the Institutional Review Board at Michigan State University and were remunerated at a rate of $15 per hour.

#### Repetitive TMS (rTMS): task and procedure

Each TMS experiment comprised of three sessions that were completed on separate days: one thresholding/practice session and two TMS sessions. A brain area was targeted unilaterally in each experiment.

We used the baseline and attention tasks from the fMRI experiment with the following modifications: (1) the staircase was specified to maintain a hit rate of 80%; (2) each run contained 100 trials; (3) to equate the duration of a block with that of the stimulation protocol (600 s), the dot fields were displayed for 3.7 s and the ITIs were changed to 1.35 s on 40% of trials, 1.8 s on 30% of trials, 2.25 s on 20% of trials, and 2.7 s on 10% of trials; (4) 70% of trials in the attention task were valid; (5) present and absent responses were always mapped to the index and middle finger, respectively; and (6) the central cross was always gray.

The thresholding/practice session occurred on a different day before the TMS session. During thresholding, subjects performed three to six runs of each task until performance was titrated to the expected level. The corresponding speedup magnitude served as the subject’s threshold and was used during TMS sessions. Ultimately, two thresholds were obtained for each subject, one for the baseline task and one for the attention task.

Each subject performed both tasks (attention and baseline) across the two TMS sessions (i.e., one task per session). During each session, subjects performed two runs of a task, one before the TMS protocol (prestimulation; see *TMS protocol*) and another immediately after (poststimulation). Task order was counterbalanced across subjects. Task performance was evaluated as the difference between the hit rate and false alarm rate. For subjects that participated in two or more experiments, only one full thresholding session was conducted. During further TMS experiments, their thresholds were simply updated by performing 50–100 thresholding trials immediately before their prestimulation run.

#### Sham TMS: task and procedure

The sham TMS experiment comprised of two sessions that were completed on separate days. In each session, subjects first performed 50–100 staircase trials to titrate performance to the expected level. The corresponding speedup magnitude served as their threshold and was used in subsequent blocks. Subjects then performed two blocks of the attention or baseline task separated by 10 min of sham TMS (see TMS protocol); task order was counterbalanced across subjects. We opted to combine the thresholding and stimulation sessions into a single session to exacerbate any potential fatigue effects.

#### TMS protocol

Three ROIs were targeted unilaterally for stimulation, left and right IPS1, and right MT+, in three separate experiments. IPS1 was topographically defined in individual subjects using the retinotopic mapping procedure while MT+ was defined using the MT+ localizer. In a given experiment, a ROI was overlaid on the corresponding T1-weighted anatomic MR image for each subject, and its centroid (visually determined) served as the target site. We used frameless stereotaxy (Brainsight 2, Rogue Research) in conjunction with a Polaris infrared positioning system (Northern Digital, Waterloo, Canada) to precisely place the coil over the target site; the coil was positioned with its handle ∼45° from the midsagittal axis. During sham TMS, the coil was centered on the mid-sagittal plane but its face was rotated 90° away from the subject’s head; thus, no cortical area received any direct stimulation.

Ten minutes of 1-Hz rTMS (600 pulses) was delivered to the target site using a Magstim Rapid^2^ stimulator and a Magstim double 70 mm air film coil (The Magstim Company). Stimulation was delivered at a fixed intensity of 70% of maximum stimulator output. This stimulation intensity was chosen because several studies have found attentional effects when stimulating the parietal cortex at similar intensities (Hilgetag et al., 2001; Thut et al., 2005; Morris et al., 2007; Schenkluhn et al., 2008; Szczepanski and Kastner, 2013). We did not use motor threshold to determine stimulation intensity because it is not necessarily a reliable index of excitability in nonmotor cortical areas (Stewart et al., 2001; Robertson et al., 2003). For this reason, and to limit the length of the experiment and the total number of TMS pulses subjects received, motor threshold was not assessed.

## Results

### Behavior in the scanner: attention task

Subjects performed a speed detection task during which they were cued to selectively attend to one of two overlapping dot fields, one rotating CW and another rotating CCW (Fig. 1*A*). On each trial, a speedup (target) occurred in either the cued direction (valid, 80% of trials) or in the uncued direction (invalid, 20% of trials) and subjects were instructed to report whether or not they perceived the speedup in the cued direction. The magnitude of the speedup was controlled by an adaptive staircase that maintained the hit rate at 65%. This 4:1 validity ratio and intermediate performance level allowed a sufficient number of incorrect (miss) trials to be collected while ensuring that performance would benefit from valid cues. On average, we obtained 95 correct and 48 incorrect trials for each subject.

**Figure 2.**
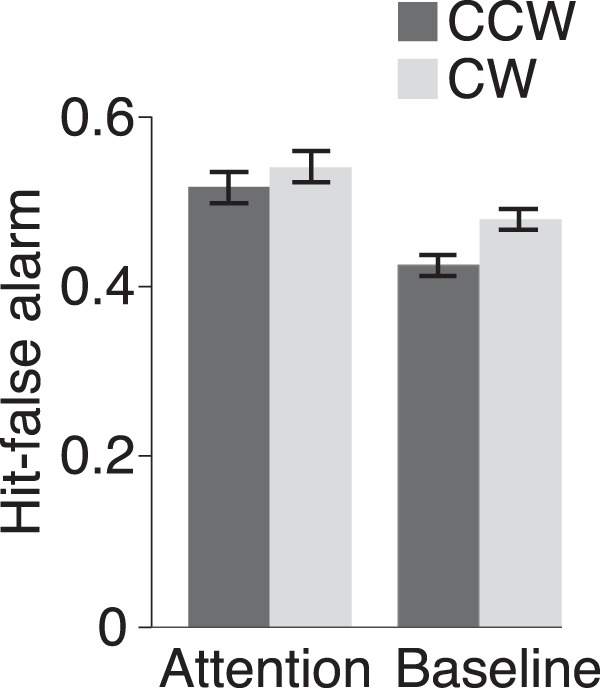
Behavioral results in the scanner. Error bars indicate ± within-subject SEM following the method of Cousineau (2005).

Consistent with our expectation, subjects successfully used the cue. The speed increment was detected near the expected hit rate (∼65%) and the false alarm rate was low (<15%). Additionally, the difference between hit and false alarm rates (i.e., hit-false alarm) revealed no difference in the ability to attend to either direction (*t*_(11)_ = 0.65, *p* = 0.53^a1^; Fig. 2). All statistics are summarized in Table 1.

**Table 1.** Statistics table

Line	Data/dependent variable	Type of test	Statistic	Confidence
	Results: behavior in scanner			
a1	Hit – false alarm	Paired *t* test (CW vs CCW)	*t = 0.65; DoF = 11;*	*p* = 0.53; CI = -0.10/0.057
a2		Paired *t* test (CW vs CCW)	*t = 2.18; DoF = 11;*	*p* = 0.052; CI = -0.11/0.0004
	Results: fMRI			
c1	BOLD response amplitude by condition	Two-way ANOVA (accuracy × direction)	*F* = 5.51; DoF = (1,11)	*p* = 0.039; general η^2^ = 0.014
c2		Two-way ANOVA (accuracy × direction)	*F* = 8.30; DoF = (1,11)	*p* = 0.015; general η^2^ = 0.018
d1	Correlation coefficients by accuracy condition (correct, incorrect)	Paired *t* test	*t = 3.21; DoF = 11;*	*p* = 0.008; CI = 0.031/0.17
d2		Paired *t* test	*t = 3.30; DoF = 11;*	*p* = 0.007; CI = 0.036/0.18
d3		Paired *t* test	*t = 3.71; DoF = 11;*	*p* = 0.004; CI = 0.061/0.24
d4		Paired *t* test	*t = 2.62; DoF = 11;*	*p* = 0.024; CI = 0.024/0.27
d5		Paired *t* test	*t = 2.60; DoF = 11;*	*p* = 0.025; CI = 0.016/0.19
d6		Paired *t* test	*t = 2.20; DoF = 11;*	*p* = 0.049; CI = 0.0002/0.20
e1	Classification accuracies by accuracy condition (correct, incorrect)	Paired *t* test	*t = 2.34; DoF = 11;*	*p* = 0.039; CI = 0.0028/0.093
e2		Permutation test (correct vs null)		*p* = 10^−3^
e3		Permutation test (incorrect vs null)		*p* = 10^−4^
	Results: TMS			
f1	Hit – false alarm	Paired *t* test (pre- vs poststimulation)	*t = 3.18; DoF = 11;*	*p* = 0.009; CI = 0.025/0.14
f2		Paired *t* test (pre- vs poststimulation)	*t = 0.12; DoF = 11;*	*p* = 0.91; CI = -0.069/0.076
f3		Paired *t* test (pre- vs poststimulation)	*t = 3.36; DoF = 11;*	*p* = 0.006; CI = 0.034/0.16
f4		Paired *t* test (pre- vs poststimulation)	*t = 0.57; DoF = 11;*	*p* = 0.58; CI = -0.044/0.075
f5		Two-way ANOVA (task × stimulation period)	*F* = 9.40; DoF = (1,11)	*p* = 0.011; general η^2^ = 0.018
f6		Two-way ANOVA (task × stimulation period)	*F* = 5.28; DoF = (1,11)	*p* = 0.042; general η^2^ = 0.010
f7		Paired *t* test (pre- vs poststimulation)	*t = 2.82; DoF = 11;*	*p* = 0.017; CI = 0.021/0.17
f8		Paired *t* test (pre- vs poststimulation)	*t = 2.26; DoF = 11;*	*p* = 0.045; CI = 0.0016/0.11
f9		Two-way ANOVA (task × stimulation period)	*F* = 11.62; DoF = (1,11)	*p* = 0.006; general η^2^ = 0.13
f10		Two-way ANOVA (task × stimulation period)	*F* = 0.90; DoF = (1,11)	*p* = 0.36; general η^2^ = 0.009
f11		Paired *t* test (pre- vs poststimulation)	*t = 1.60; DoF = 11;*	*p* = 0.14; CI = -0.14/0.021
f12		Paired *t* test (pre- vs poststimulation)	*t = 0.34; DoF = 11;*	*p* = 0.74; CI = -0.073/0.053

### Behavior in the scanner: baseline task

In the same scanning session, subjects performed another speed detection task during which they were presented with a single dot field (Fig. 1*B*). On each trial, the dot field rotated either CW or CCW and a speedup occurred in 70% of trials. On the remaining 30% of trials, no speedup occurred. The magnitude of the speedup was controlled by an adaptive staircase that maintained the hit rate at 50%. This experimental design kept subjects engaged in the task and allowed us to obtain a sufficient number of correct rejection trials (when no speedup was presented and subjects correctly reported its absence) for subsequent analyses. On average, we obtained 51 correct rejection trials for each subject.

Behavioral performance indicated that subjects were successful at detecting the speedup in both directions. The hit rate was near the expected performance level and the false alarm rate was low (<5%). The difference in performance during CW and CCW motion was marginally significant (*t*_(11)_ = 2.18, *p* = 0.052^a2^), suggesting that it was easier to detect a CW target (Fig. 2). This difference was unexpected. However, we note that any performance difference in the baseline task should not affect our fMRI analyses because only correct rejection trials (i.e., trials without a target) were used.

### Attention and baseline tasks modulate BOLD response in occipital and frontoparietal areas

To identify cortical areas that were modulated by the tasks, we performed a subject-based deconvolution analysis. Voxel-wise deconvolved responses were computed for each condition and the amount of variance in the time course accounted for by the deconvolution model (*r* ^2^) represented the extent of task modulation. Group-averaged *r* ^2^ maps were visualized in the PALS atlas space using spherical registration (for details, see Materials and Methods).

**Figure 3.**
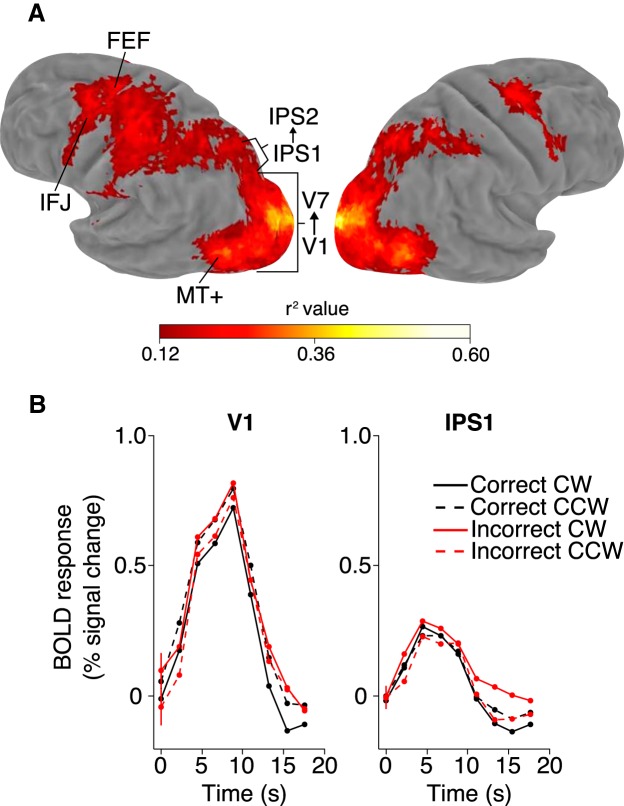
Univariate results. ***A***, Group *r* ^2^ map of the attention task shown on an inflated Caret atlas surface. The approximate locations of retinotopically-defined (V1-7, MT+, IPS1, and IPS2) and task-defined (FEF and IFJ) areas are indicated by lines. ***B***, Mean BOLD response in the attention task from two ROIs (V1 and IPS1). The error bar on the first time point is the average ± within-subject SEM across all time points.

At the group-level, a network of frontoparietal areas, as well as occipital visual areas, showed significant modulation by the both the attention (Fig. 3*A*) and baseline (data not shown) tasks. This overall pattern of activation was similar to findings from many previous studies of attentional control (Kastner and Ungerleider, 2000; Corbetta and Shulman, 2002). Active areas in the occipital and parietal cortices overlapped with areas defined via retinotopy. Additionally, we defined two frontal ROIs (FEF and IFJ) in each subject using their *r* ^2^ map of the attention task. FEF was defined near the junction of the precentral and superior frontal sulcus and IFJ was defined near the junction of the precentral and inferior frontal sulcus. These two regions appeared contiguous on the group-averaged map, but they formed distinct clusters in individual subject maps. In each subject, we obtained 11 ROIs: V1, V2, V3, V3A/B, V4, MT+, V7/IPS0, IPS1, IPS2, FEF, and IFJ. No laterality was observed in the results for each area, therefore we averaged the data from corresponding areas across the two hemispheres.

### BOLD amplitude does not vary with task performance

We observed robust task-related BOLD responses in all of our ROIs and, for illustrative purposes, we plotted the response of V1 and IPS1 (Fig. 3*B*). The BOLD response to all conditions in the attention task (accuracy crossed with cued direction, CW and CCW) peaked between 4.4 and 8.8 s after trial onset (time points 3–5) and was very similar between conditions. To quantify this observation, we computed the average response amplitude for each condition and conducted a two-way repeated-measures ANOVA (two accuracy conditions × two attended directions). The analysis revealed a main effect of direction in IPS2, response amplitude was larger for CW than CCW attention (*F*_(1,11)_ = 5.51, *p* = 0.039^c1^), and an interaction in V1, when correct, response amplitude was larger for CCW than CW attention; when incorrect, the pattern was reversed (*F*_(1,11)_ = 8.30, *p* = 0.015^c2^). Importantly, the main effect of accuracy was nonsignificant in all ROIs (all *p* > 0.2), therefore, overall BOLD amplitude did not vary with performance. This result is thus inconsistent with an interpretation that performance variation in the attention task was caused by fluctuations of general behavioral state (e.g., fatigue or vigilance), as such effects are known to be reflected in overall BOLD amplitude variations (Boly et al., 2007; Esterman et al., 2013).

### Average neural patterns for prioritized features in occipital and frontoparietal areas vary with task performance

**Figure 4.**
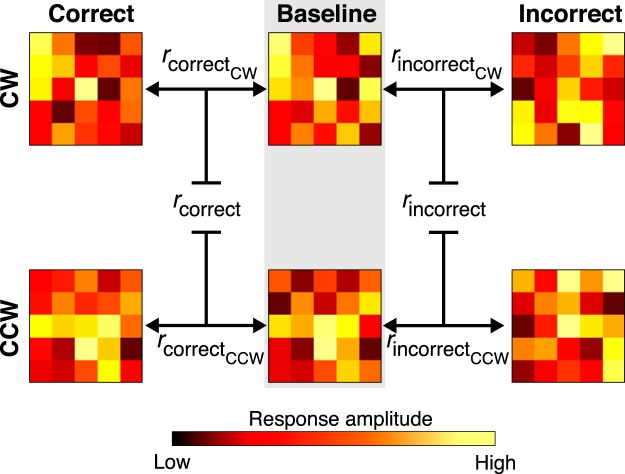
Schematic of the correlation analysis. Each matrix represents the spatial pattern of response amplitudes from voxels within a ROI (amplitude is color coded according to the scale bar at the bottom). The middle column of matrices (shaded area labeled “baseline”) illustrates the baseline neural response pattern to each direction (CW and CCW) during the baseline task. The other two columns illustrate the neural response pattern to an attended motion direction during correct and incorrect trials during the attention task. The black double-sided arrows between matrices represent the correlations that were calculated (Pearson’s *r*) and the bounded lines represent the averaging of correlation coefficients across directions to obtain an overall index of attentional priority quality for correct and incorrect trials.

To examine a possible relationship between attentional priority and task performance, we used a correlation analysis to compare the spatial pattern of neural activity when subjects were correct versus incorrect in the attention task (Fig. 4). Voxel-wise BOLD responses were used to obtain spatial patterns of neural activity for attended and isolated features (CW and CCW motion). Separate Attention patterns were constructed for CW and CCW-cued trials in the attention task, and baseline patterns were constructed with voxel responses during correct rejection trials in the baseline task. The response to correct rejections was used because there was neither a physical nor perceived target during these trials, which made them suitable for isolating feature-specific neural responses, uncontaminated by target-related responses. We used the correlation between baseline and attention patterns to index the quality of attentional priority, with low (high) correlations reflecting weak (strong) feature selection.

**Figure 5.**
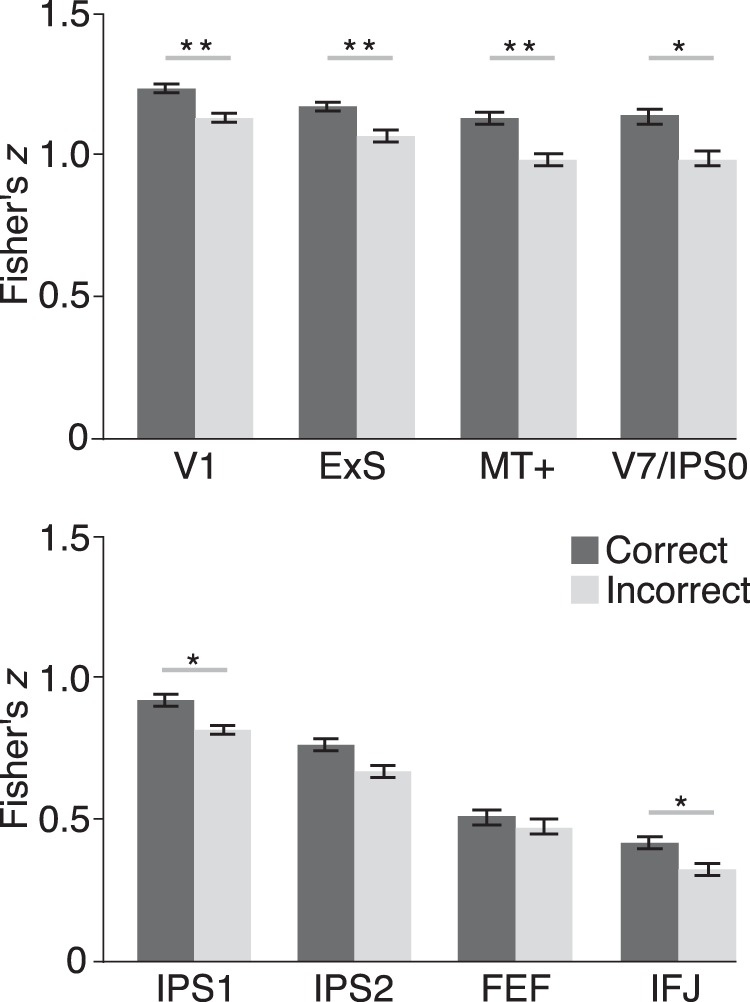
Average neural patterns for prioritized features in occipital and frontoparietal areas vary with task performance. Group-average Fisher-transformed correlation coefficients (averaged across motion directions) are shown, which reflect the similarity of neural patterns of activity between the attention and baseline tasks for correct and incorrect trials. The ExS label represents extrastriate visual areas. Error bars are ± within-subject SEM following the method of Cousineau (2005). Asterisks indicate the significance level in paired *t* tests (**p < 0.01, *p < 0.05).

We conducted planned comparisons between the quality of attentional priority on correct and incorrect trials within each ROI (Fig. 5). Because all visual areas showed similar results and because we are primarily interested in frontoparietal areas’ role in attentional control, we aggregated data from extrastriate visual areas (abbreviated to ExS in figures) by averaging the correlation coefficients across V2, V3, V3A/B, and V4. We kept MT+ separate as there is a strong a priori link between MT+ activity and motion processing. We found that Baseline and Attention patterns were more correlated for correct than incorrect trials in V1 (*t*_(11)_ = 3.21, *p* = 0.008^d1^), extrastriate regions (*t*_(11)_ = 3.30, *p =* 0.007^d2^), MT+ (*t*_(11)_ = 3.71, *p* = 0.004^d3^), V7/IPS0 (*t*_(11)_ = 2.62, *p =* 0.024^d4^), IPS1 (*t*_(11)_ = 2.60, *p* = 0.025^d5^), and IFJ (*t*_(11)_ = 2.20, *p* = 0.049^d6^). These results demonstrate that the spatial pattern of neural activity within these cortical areas encodes a more veridical representation of the attended feature during correct trials. The observed neural-behavioral correlates in posterior parietal and inferior frontal areas support their role in encoding attentional priority for features, whereas the analogous effects in visual areas likely reflect attentional modulation due to feedback. In the following, we sought converging evidence with a classification approach that provided another measure of pattern similarity.

### Feature coding in posterior parietal cortex (PPC) tracks trial-by-trial fluctuations in task performance

The correlation analysis above used the average neural response across trials; yet, attentional control likely fluctuates across individual trials, leading to different behavioral outcomes. Therefore, a trial-by-trial assessment would reveal cortical areas that consistently encode the attended feature and facilitate task performance.

**Figure 6.**
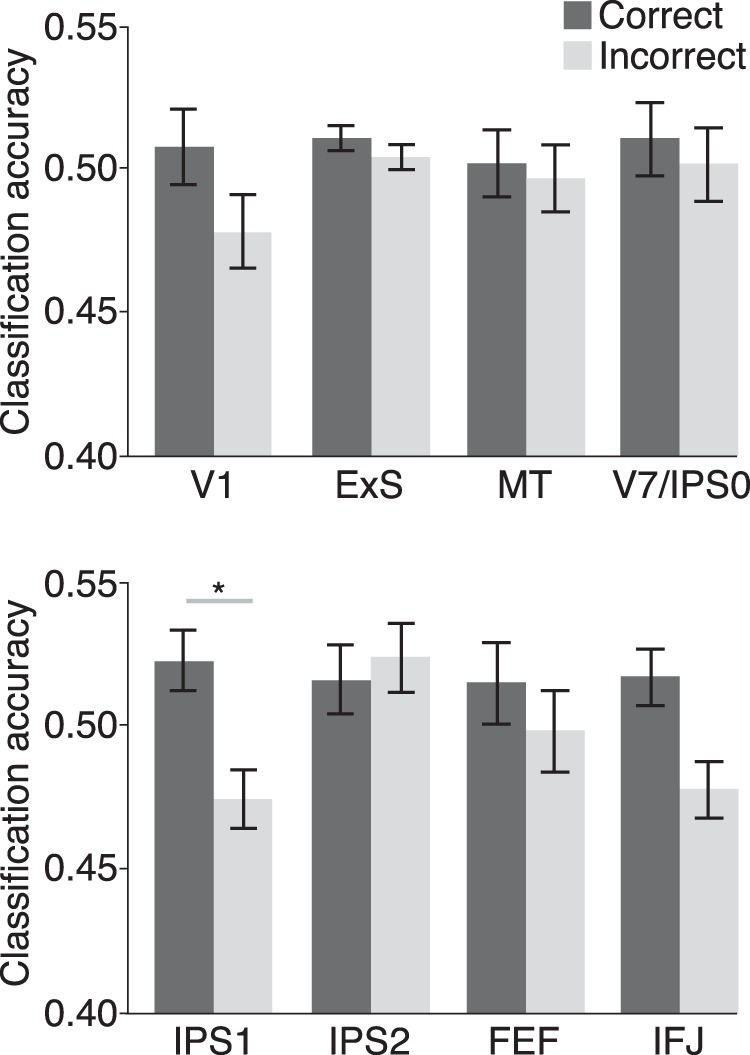
Feature coding in PPC tracks trial-by-trial fluctuations in task performance. Group-average classification accuracies are shown and plotting conventions are the same as Figure 5.

A classification approach was used to assess feature representation on individual trials. A linear classifier was trained with correct rejection trials from the baseline task to discriminate between CW and CCW motion. Then, the classifier was tested on how well it decoded the attended direction in individual correct and incorrect trials of the attention task. For each ROI, classification accuracy for correct and incorrect trials were compared (Fig. 6), revealing that the attended direction was better discriminated for correct than incorrect trials in IPS1 (*t*_(11)_ = 2.34, *p* = 0.039^e1^). Moreover, by constructing a null-distribution that characterized chance-level classification performance, we found that the attended feature was discriminated above chance for correct trials (*p* = 10^−3^) and below chance for incorrect trials (*p* = 10^−4^). These results indicate that IPS1 consistently encodes a more veridical representation of the attended feature during correct than incorrect trials. The below-chance classification for incorrect trials is somewhat unexpected; this could suggest that errors were partly due to subjects attending to the uncued feature on those trials, which would lead to opposite neural response patterns for the training and test datasets. Overall, both our correlation and classification approaches provide converging evidence of IPS1’s role in the maintenance of attentional priority.

### PPC is necessary for feature-based attention

Because our multivariate analyses indicated that neural activity patterns in IPS1 correlate with feature selection and task performance, we sought to assess whether it is causally involved in feature-based attention. Subjects performed the attention and baseline tasks while neuronavigated rTMS was used to disrupt neural processing unilaterally in left or right IPS1. Stimulation was centered on each retinotopically-defined ROI, but given the relatively small size of these cortical areas compared to the estimated spatial extent of TMS stimulation (Walsh and Pascual-Leone, 2003), we refer to the area stimulated as the PPC.

**Figure 7.**
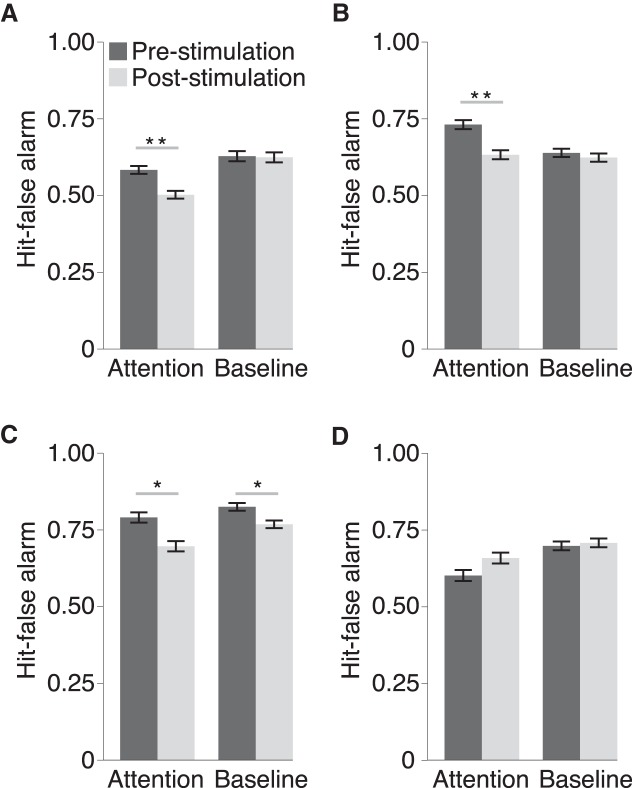
Results of the TMS experiments. Each panel presents the results for stimulation centered on (***A***) left IPS1, (***B***) right IPS1, (***C***) right MT+, and (***D***) sham TMS. Plotting conventions are the same as Figure 5.

We reasoned that because feature-based selection was necessary in the attention task, but not the baseline task, disruption of areas primarily representing attentional priority would impair performance in the attention task and leave performance in the baseline task largely unchanged. With this rationale, we conducted two separate experiments in which rTMS was applied unilaterally to left or right PPC. Before TMS sessions, speedup magnitude thresholds for the attention and baseline tasks were obtained for each subject such that hit rates were equated. Then, on a separate day, subjects performed two blocks of either task, separated by 1-Hz rTMS. After stimulation of left PPC (Fig. 7*A*), performance was significantly impaired in the attention task (*t*_(11)_ = 3.18, *p* = 0.009^f1^) but not the baseline task (*t*_(11)_ = 0.12, *p* = 0.91^f2^). Similarly, stimulation of right PPC (Fig. 7*B*) impaired performance in the attention task (*t*_(11)_ = 3.36, *p* = 0.006^f3^) but not the baseline task (*t*_(11)_ = 0.57, *p* = 0.58^f4^). Consistent with these planned comparisons, a two-way repeated measures ANOVA (two tasks × two stimulation periods) revealed a significant interaction effect for left (*F*_(1,11)_ = 9.40, *p* = 0.011^f5^) and right (*F*_(1,11)_ = 5.28, *p* = 0.042^f6^) parietal stimulation sites. Thus, these results support a causal role of PPC in the maintenance of attentional priority that mediates feature selection.

### Neurodisruption of MT+ does not selectively impair feature-based attention

Because our fMRI correlation analysis also revealed performance correlates in visual areas, we conducted a third TMS experiment where we stimulated right MT+ to further dissociate visual areas from control areas of attention (Fig. 7*C*). Given its critical role in visual motion processing (Newsome and Paré, 1988), we reasoned that neurodisruption of MT+ should lead to equivalent decrements in performance for the attention and baseline tasks because both rely on basic motion processing. Consistent with this prediction, rTMS impaired performance in the attention (*t*_(11)_ = 2.82, *p* = 0.017^f7^) and baseline task (*t*_(11)_ = 2.26, *p* = 0.045^f8^). This was verified by a two-way repeated measures ANOVA that revealed a main effect of stimulation period (*F*_(1,11)_ = 11.62, *p* = 0.006^f9^) and no interaction effect (*F*_(1,11)_ = 0.90, *p* = 0.36^f10^).

### General behavioral state does not explain performance decrements due to rTMS

A potential explanation for the performance decrements observed in the above TMS experiments might relate to variations in general behavioral state (e.g., fatigue or vigilance). In particular, impaired performance post stimulation could have arisen simply because subjects were fatigued after completing their first block of trials, and this fatigue might be particularly severe for the attention task. To assess this possibility, we performed a fourth experiment in which no cortical area was directly stimulated. Instead, the coil face was oriented 90° away from the scalp (sham TMS). In addition, we exacerbated potential fatigue effects by combining thresholding and TMS sessions into a single session. Thus, subjects first completed one to two blocks of staircase trials, and then immediately performed the pre- and post-stimulation blocks, separated by sham TMS. We reasoned that if fatigue contributed to our TMS effects, then performance would be impaired in the post-block even without brain stimulation. Our results reject this hypothesis (Fig. 7*D*): performance was unchanged between pre- and postblocks in the attention task (*t*_(11)_ = 1.60, *p* = 0.14^f11^) and baseline task (*t*_(11)_ = 0.34, *p* = 0.74^f12^). Thus, fatigue cannot account for the observed effects of PPC and MT+ stimulation.

Overall, we found that applying rTMS to PPC produced a selective deficit in the attention task, demonstrating its specific role in attentional selection. This was in contrast to MT+ stimulation that produced a deficit in both tasks, demonstrating its general role in motion processing. Thus, our results provide converging evidence that PPC, and IPS1 in particular, is causally involved in the maintenance of attentional priority.

## Discussion

In this study, we examined how neural activity in dorsal frontoparietal areas is related to behavioral performance in a feature-based attention task. We found that attending to a feature (motion direction) produced spatial patterns of neural activity in frontal, parietal, and visual areas that better resembled those of an isolated feature (evoked by a single motion direction) during correct than during incorrect trials. On a trial-by-trial basis, this pattern congruency effect was uniquely found in IPS1, as revealed by pattern classification analyses. Additionally, we found that rTMS centered on IPS1 led to a performance impairment in the attention but not the baseline task, whereas rTMS to MT+ led to equivalent impairments in both tasks. Finally, sham TMS did not change performance in either task, ruling out the influence of general behavioral state on task performance. These results reveal that frontoparietal areas maintain attentional priority that facilitates the selection of visual features, and in particular, PPC, including IPS1 plays a causal role in such selection.

Previous studies have provided indirect evidence that frontoparietal neural activity is related to the control of feature-based attention (Serences and Boynton, 2007; Liu et al., 2011; Ester et al., 2016). However, whether these neural signals mediate task performance and how such signals represent the attended feature are unclear. Here, we addressed both issues by examining the relationship between patterned neural activity and task performance, as well as the consequence of neural perturbation on task performance.

The baseline task provided a measure of the neural pattern evoked by each motion direction without selective attention. To the extent that subjects successfully attended to the cued direction in the attention task, the observed neural pattern should resemble the baseline pattern for that direction and facilitate performance in a difficult threshold-level detection task. Indeed, we found such an effect in visual areas including V1 and frontoparietal areas (V7/IPS0, IPS1, and IFJ). In these areas, the correlation between the attention pattern and the baseline pattern was greater for correct than incorrect trials, suggesting that feature-based attention operates by biasing the population activity toward that evoked by the feature alone. These results are consistent with the finding that neuronal tuning in V4 is shifted to the attended feature (David et al., 2008) as well as with the finding that attention shifts fMRI voxel’s semantic category representations during visual search in natural movies (Çukur et al., 2013). Our results are complementary to these previous findings and go beyond them by demonstrating that attention-induced shifts in neural population activity are functionally significant in that they correlate with task performance.

We also used a pattern classification approach to assess the relationship between neural activity patterns and task performance. Because the classification approach uses single-trial data, this allows us to examine how trial-by-trial variations in neural activity contribute to attentional selection. This analysis showed that the pattern difference between the two features, the information extracted by the classifier, was more aligned between the baseline and the attention tasks on correct trials. This result suggests that to the extent that the brain could rely on the same discriminative information between the two features when they were presented alone, successful attentional selection can be achieved when they were presented together in competition. We note that we only found this result in IPS1, while the correlation analysis found neural correlates of behavioral accuracy in multiple cortical areas. The difference in results could be due, in part, to the use of mean patterns across trials in the correlation analysis that reflect an overall effect of attention, and the use of single-trial BOLD responses in the classification analysis, which are sensitive to trial-level fluctuations. Although, in principle, such fluctuations should manifest both at the source and the destination of attentional modulation, it is plausible that neural conduction would introduce additional noise at the destination relative to the source. If so, it would be easier to detect a performance-based effect at the source region than the destination region. Thus, our classification results indirectly support the notion that IPS1 contains the source of attentional control that modulates visual areas during feature-based selection. In addition, single-trial neural patterns used in the classification analysis are likely to be quite noisy, which could make it difficult to achieve reliable pattern separation. Indeed, the overall classification accuracy in our results was rather low, presumably limited by the noisy estimate of the trial-level responses and the limited size of the training dataset. Although the effect in classification accuracy was somewhat weak, it provided additional evidence that IPS1 is an important cortical area in shaping attentional selection, and guided our selection of PPC as a stimulation target during TMS.

To obtain converging evidence, we used rTMS to disrupt local neural processing in PPC to test its causal role in feature-based attention. We reasoned that if PPC is causally involved in attentional selection, neurodisruption should produce behavioral deficits in the attention task and not the baseline task. Importantly, performance in both tasks was titrated to be at equivalent levels with adaptive staircase methods. Therefore, general task difficulty cannot explain any differential effect produced by neurodisruption. Our results support this prediction: disrupting PPC produced a behavioral deficit in the attention task but not in the baseline task. We dissociated this selective top-down attentional impairment from bottom-up motion processing by stimulating MT+, a region with strong a priori links to bottom-up processing of visual motion. Because each task relies on basic motion processing, we reasoned that neurodisruption should produce equivalent behavioral deficits in both tasks. Consistent with this prediction, disrupting MT+ yielded equivalent behavioral deficits in both tasks. Finally, we dissociated the effects of neurodisruption from variations in general behavioral state (e.g., fatigue or vigilance) with sham TMS and protracted experimental sessions. We reasoned that if variations in task performance are due to fatigue, behavioral deficits should be observed in both tasks even without neurodisruption. Our results reject this hypothesis as performance was unchanged with sham TMS. Overall, these findings demonstrate that neural activity in PPC, and in particular, IPS1, is causally and specifically involved in the control of feature-based attention.

Our TMS results apparently contradict those of a previous study that found that stimulation of anterior, but not posterior, IPS impaired performance in a feature-cued visual search task (Schenkluhn et al., 2008). However, there are some critical differences between the TMS protocol used in the two studies. They used an online protocol to deliver brief TMS pulses right after the cue, which was presumed to only affect neural activity during the cue period. In contrast, the effects of our offline protocol have been shown to persist during the test block (Hilgetag et al., 2001; Thut et al., 2005; Zanto et al., 2011). Therefore, it is possible that anterior IPS plays a role in cue processing (e.g., setting the task goal), whereas posterior IPS plays a critical role in actively maintaining the selection of a goal-relevant feature during stimulus presentation. Future studies could determine the time course of relevance for these areas by using TMS to disrupt anterior and posterior IPS at different times during cue and stimulus presentation.

Our converging fMRI and TMS results highlight the role of the PPC in controlling feature-based attention by demonstrating a strong behavioral correlate between parietal activity and task performance and its causal role in guiding behavior. Considering the strong support of this area in the control of spatial attention (Bisley and Goldberg, 2010; Buschman and Kastner, 2015), the overall results suggest that parietal areas contain domain-general attentional control signals for both spatial and nonspatial attention. Our fMRI correlation results also revealed a neural correlate of task performance in IFJ. IFJ has been suggested to be a shared node between the dorsal and ventral attentional control network (Corbetta et al., 2008) and recent studies have demonstrated its role in object-based attention and working memory (Zanto et al., 2011; Baldauf and Desimone, 2014), which are closely related to feature-based attention. The overall results suggest that IFJ and PPC represent attentional priority of features by maintaining population neural activity similar to that evoked by those features alone. These neural signals could serve as attentional templates, which have been proposed to control the deployment of feature-based attention in theoretical models (Wolfe, 1994; Desimone and Duncan, 1995). We note, however, that the overall magnitude of correlation in IFJ is low and it also did not exhibit performance-related effects in the classification analysis. Therefore, its role in feature-based selection might be weaker than object-based selection or working memory. In addition, other areas in the frontoparietal network did not exhibit any fMRI correlate of performance. In particular, fMRI pattern similarity in IPS2 and FEF did not vary with task performance although previous studies have shown that neural signals in these areas can be used to decode the attended feature (Liu et al., 2011; Liu and Hou, 2013; Ester et al., 2016). It is conceivable that other types of control signals, the ones that do not necessarily resemble those that process the original features, also participate in attentional selection. For example, these areas in the network could encode more abstract, perhaps categorical, information that guides attentional selection. The precise role of individual cortical areas in coordinating attentional selection awaits further investigation.

More broadly, our results are also informative regarding the general role of PPC in visual processing. Traditionally, parietal cortex has been associated with visuospatial and visuomotor processing (Mishkin and Ungerleider, 1982; Andersen and Buneo, 2002). However, more recent work has demonstrated neural selectivity to many nonspatial properties in this cortical area, such as simple features (Sereno and Maunsell, 1998; Toth and Assad, 2002), arbitrary categories (Freedman and Assad, 2006; Fitzgerald et al., 2011), and even abstract identity information (Jeong and Xu, 2016). Our results are thus consistent with this emerging view of nonspatial representation in parietal cortex and further suggest that such nonspatial representations can facilitate the selection of behaviorally relevant visual features.

## Synthesis

January 15, 2018

Dear Dr. Li,

Thank you for your editorial work regarding our manuscript submitted to eNeuro. We are glad to see the reviewers generally found our results interesting and informative. Both reviewers have made insightful comments regarding the rationale of our behavioral manipulations, and how our fMRI results were presented. In this revision, we have further clarified some methodological and result-related issues. As a result, we feel our manuscript is much improved and we want to thank the reviewers for the help.

Below, we address the reviewers' comments in detail. The revised portion of the manuscript is highlighted with a green font for ease of re-review.

Thank you very much for your consideration.

Editor's comments (comments in blue and our responses in black)

Synthesis Statement for Author (Required):

Both expert reviewers think that the ms addressed an important question, i.e., the neural mechanism for feature-based attention, and used multiple approaches to obtain converging evidence. However, both reviewers also think there are specific issues that need to be addressed before the ms can be accepted for publication. Note that both reviewers also pointed out the “testing of the software” is confusing as the mrTools package used by the authors is extensively used in the field. I list the reviewers' detailed comments below.

[Response] We are sorry but we are not quite sure what “testing of the software” refers to. However, we have addressed each point raised by the reviewers, including some analytical details. We and other labs use mrTools extensively in analyzing fMRI data and we strive to be as clear as possible in describing the analytical procedures. We hope you and the reviewers found our revised description of the data analysis adequate.

Reviewer 1:

This manuscript presents a study using fMRI and TMS to investigate the roles of different cortical areas in mediating behavioral performance during feature-based attention task in the human brain. The fMRI results showed that posterior parietal cortex (IPS1) is the key area that maintain attentional priority in feature-based attention. The TMS results causally confirmed this conclusion by comparing the interference effects in posterior parietal cortex and early sensory area.

This is a well designed and conducted study. The methods and results are properly documented in the manuscript. Overall, the finding will contribute to the existing literature and deepen our understanding about the neural correlates of task performance in feature-based attention in the human brain. I have some comments as listed below, and these comments can be addressed by adding extra discussion and/or analyses.

1.My main concern is about the MVPA analysis. Serval issues related to this analysis deserve extra caution. First, when selected the voxels for each ROI, r^2 values from the attention task (i.e., the testing dataset) were used for ranking the voxels. Apparently, using the baseline task (i.e., the training dataset) to select the voxels will be the optimal way to maximize the independency between training and test in cross-validation.

[Response] Thank you for this point. We agree that ranking voxels based on the baseline task (i.e., the training dataset) will maximize the independency during cross-validation. To address this issue, we repeated our classification analysis with a range of voxels (5-145), ranked by r2 values from the baseline task. Below, we show group-averaged classification accuracies for representative visual (V1), parietal (IPS1), and frontal (IFJ) areas, with shaded regions representing the standard error of the mean. In IPS1, the attended feature (CW or CCW) is better discriminated during correct than incorrect trials with as few as 35 voxels and up to 145 voxels, with the difference reaching statistical significance when the top 55 voxels are used (t(11) = 2.34; p=0.039). In contrast, visual and frontal regions do not show any difference between correct and incorrect trials with any number of voxels. We acknowledge that the difference in IPS1 is numerically small and is not highly significant in a statistical sense. Nonetheless, the consistent difference between correct and incorrect trials in IPS1, and the lack thereof in other areas, do hint at a functional difference between these areas. So we felt this finding still provides further converging evidence of IPS1's role in feature-based attention (and we now further acknowledge this somewhat weak effect, see below).

In the manuscript, we now use the classification results obtained with the top 55 voxels in the baseline task. We have changed the Methods (lines 286-288) and Results (lines 507-508, 510) to reflect this change.

Second, in lines 269-270, it says that “averaging the time series between the time of peak response, as defined by a subject's response profile to the shortest possible trial duration (8.8 s)”. Some information is missing here: between the time of peak response and “...”.

[Response] We apologize for the confusion here. For our classification analysis, we averaged the time series between the time of peak response, as defined by a subject's response profile (between the 3rd and 5th time points, respectively), to the shortest possible trial duration (8.8 s; 5th time point). In the manuscript, we have now added a comma between “profile” and “to” (line 290) in order to clarify this.

Third, the MVPA results were not very promising. The only significant difference came from IPS1. However, for IPS1, the classification accuracy was very close to chance level (i.e., 50%) for the correct condition. The significance was mainly due to the much below chance-level accuracy for the incorrect condition. The below chance-level classification accuracy could have different interpretations, one of which is that the training dataset and test dataset have opposite response patterns. Therefore, I would suggest the authors to emphasize less about the implication of the MVPA analysis and just use it as a guidance to the TMS stimulation.

[Response] We agree that our classification results are not very robust, and the below chance classification for incorrect trials could be due to opposite response patterns for training and test datasets. We made the conjecture that on those trials subjects might attend to the wrong feature, which will indeed lead to opposite response patterns, as suggested. We have added this idea to the Results (lines 514-515). Furthermore, following your suggestion, we address the somewhat weak classification in the Discussion (lines 625-628) and clarify that these results were used to guide our selection of IPS1 as our stimulation target during TMS.

2.Line 221, it was not clear why false alarm is used as a single regressor, but not divided into two for CW and CCW conditions.

[Response] Thank you for raising this point. Due to the low proportion of invalid trials in the attention task, false alarms were scarce (5±5 false alarms across subjects; 7 subjects had < 3 false alarms). Therefore, we did not have enough trials to create CW and CCW regressors in all subjects. We have added text in lines 235-237 to clarify this point.

3.Line 408, p=0.052 is marginal significance. Fig 2 does show this trend.

[Response] Thank you for pointing this out. We have acknowledged this marginal significance. However, because only correct rejection trials were used in the fMRI analysis, we do not see this as problematic. We added text on lines 428-432 to discuss this issue.

4.Line 319, “27 subjects participated in the TMS experiments, with 12 participating in each 320 experiment”. There were 24 subjects in total?

[Response] We appreciate this comment. There were 27 subjects and 3 TMS experiments, with some subjects participating in more than one experiment. We have added more text that details the distribution of subjects across TMS experiments (lines 339-342).

Reviewer 2:

General comments:

The authors employed a variety of fMRI analyses and TMS experiments to examine the relationships between feature-based attention and performance of a visual task and the corresponding neural substrates. They found that in multiple occipital, parietal, and frontal cortical areas, activity associated with directing attention to a visual feature (a direction of motion) predicted successful task performance when this activity was similar to activity evoked by presentation of the attended feature in isolation. In area IPS1, this relationship was evident on a trial-by-trial basis, as assessed with pattern classification analysis. Finally, application of rTMS to posterior parietal cortex selectively reduced performance in the feature attention task, while rTMS to MT+ impaired performance in both tasks. This study provides important new information about the neural basis of feature-based attention and how it affects visual performance, and the TMS experiments strengthen the paper by establishing causality.

However, there are also some issues. There are multiple differences between the attention and baseline tasks, both in task design and analysis, that should be discussed and justified. In addition, more discussion is needed concerning 1) the fact that feature-based attention was not required for the majority of the visual stimulus presentation, 2) the procedures for retinotopic mapping and ROI definition, and 3) statistical corrections for multiple comparisons.

Specific comments:

Major points:

1) The attention and baseline tasks are not always accurately described. As I understand them, the attention task requires a discrimination between which of two possible directions of motion contained the speed increase. In contrast, the baseline task is a speed increment detection task (present versus absent). Because the attention task always had a speed increment, it seems incorrect to classify trials from this task as “hits”, “misses”, and “false alarms”, as these terms are typically associated with detection, not discrimination, tasks. Also, the term “hit rate” should probably not be used to describe correct performance on valid trials in the attention task.

[Response] We apologize for the confusion. It is the case that the attention task contained a speedup on each trial; however, instead of reporting the direction that contained a speedup, subjects were instructed to report whether the speedup was “Present” or “Absent” in the cued direction. Thus, performance was contingent on the attended feature. For example, if subjects were cued to attend CW motion, then they were tasked to report whether a speedup was “Present” or “Absent” in that motion direction. Thus, when a CW speedup occurred on a CW-cued trial, a “Present” response was classified as a “hit” and an “Absent” response was classified as a “miss.” Alternatively, if a CCW speedup occurred when CW motion was cued, a “Present” response was classified as a “false alarm” and an “Absent” response as a “correct rejection.” Therefore, the attention task was designed such that subjects tracked and reported on only the cued motion direction, instead of tracking both directions and reporting which one sped up. The attention task is thus essentially a detection task. We have clarified this in the Methods (lines 117-120).

Finally, I don't think “catch trial” is the appropriate term for the trials on the baseline task in which there was no speed increment. I believe that “catch trials” usually refer to trials for which the difficulty is very low and the experimenter is confident of what the subject's response should be.

[Response] We believe that our use of “catch trials” is correct. A catch trial is defined as, “a trial in which no stimulus is presented...to estimate the individual's baseline tendency to give positive responses” (Colman, 2015). A low-difficulty trial (i.e., an easy trial) would instead be useful in estimating a subject's lapse rate, that is, their tendency to give negative responses. However, we recognize there could be potential confusions as to what these trials are supposed to “catch”. To clarify this and avoid any future confusion, we have changed “speedup” and “catch” trials to “target-present” and “target-absent” trials, respectively (lines 147 and 173).

2) There are multiple differences between the attention and baseline tasks that make comparisons between them more difficult to interpret. Why was the “hit rate” set to be different for the two tasks (65% for attention task and 50% for the baseline task) in the fMRI experiments?

[Response] There are indeed some differences between the attention and baseline task; however, these tasks were matched as closely as possible except for the critical experimental variable (the need for selective attention in one task and the lack thereof in the other task). Importantly, we did not directly compare data from the two tasks. Instead, we measured the relationship of pattern separation (between two features within a task) across two tasks--akin to a representational similarity analysis. In other words, we are really studying how neural similarity structure changes across tasks. The specific performance levels were adopted to provide sufficient number of trials for the multivariate analysis and provide appropriate incentive for performing the threshold tasks. For the baseline task, we aimed to maximize the number of correct rejections because those were our measures of baseline neural patterns (further clarified in lines 148-152). Here, we chose a low hit rate (50%) to dissuade subjects from reporting too many false positives, which would reduce correct rejections. For the attention task, we aimed to maximize both hits and misses while ensuring that subjects would still use the cue to aid performance (further clarified in lines 130-134). Though a 50% hit rate would mathematically maximize the number of hits and misses, such a low performance level could discourage subjects from using the cue, i.e., the utility of the cue would be rather limited. Thus, we opted for an intermediate hit rate (65%) in the attention task.

The fact that the attention task is a discrimination task but the baseline task a detection task results in different numbers of regressors (five for attention task; seven for the baseline task) in the fMRI analysis. Also, even though the authors only focus on valid trials in this paper, why not model invalid trials in the same way as the valid trials (CW vs. CCW and correct vs. incorrect)? There would still be different numbers of regressors in the two tasks in this case (presumably eight instead of five for the attention task), but it would at least make the analysis of the two tasks more comparable.

[Response] Thank you for this point. We used different numbers of regressors to model the fMRI time series because each task contained different numbers of relevant conditions. Again, we want to emphasize that we did not directly compare data from the two tasks (e.g., run a GLM across tasks and contrast two conditions, see the point above). In addition, in practical matters, due to the low proportion of invalid trials in the attention task, false alarms were rather scarce (5±5 false alarms across subjects; 7 subjects had < 3 false alarms). Thus, it became impractical to model these trials separately, and indeed, modeling conditions with very few trials could also affect our ability to accurately estimate the other conditions of interest. We have added text in lines 235-237 to clarify this point.

Finally, given basic differences between the tasks (detection versus discrimination), it is problematic to claim that performance was titrated to be at equivalent levels for the TMS experiments.

[Response] Because the attention and baseline tasks were both designed as detection tasks (as explained above), we believe that our titration procedure for the TMS experiments did equate performance across tasks.

3) Because the speed increment always happened at the very end of the stimulus presentation (the last 200 ms of a stimulus that was presented for 4.1 seconds), feature-based attention was not needed for the majority of the stimulus duration. In fact, the subjects could have completely ignored the stimulus until the very end. However, the analysis of fMRI signals as a function of task performance models the response to presentation of the entire stimulus. How might the results be different if subjects engaged feature-based attention during all of the stimulus presentation? This should be discussed by the authors.

[Response] Thank you for this point. We agree that constraining the speed increment to occur at the end of stimulus presentation did not guarantee that the cued direction was attended throughout. However, this is not a trivial issue with the somewhat prolonged trial duration, which is necessitated by the slowness of the BOLD signal. For example, if we had made the speed increment to occur randomly within stimulus presentation, it would still be possible for attention to disengage prematurely, that is, subjects would stop paying attention as soon as the target occurred.

One strategy we adopted to handle this issue was to extensively train our subjects prior to their imaging session. During training, subjects were instructed to maintain attention throughout the stimulus duration and all subjects reported compliance. With well-trained subjects, we expected more consistent attentional engagement during the imaging session. Nevertheless, it is still possible that attention was not directed consistently throughout a trial. However, partial and/or inconsistent attentional engagement would likely reduce the attentional signal and increase noise in neural data, such that it would be more difficult to detect differences between correct and incorrect trials. Thus, we think that if attention had been engaged more consistently throughout the stimulus duration, it would make our observed effects even stronger. We have added some text in the Methods discussing this point (lines 160-165).

4) More detailed description of the retinotopic mapping and definition of visual cortical areas should be provided. The annulus used in the main experiment had 2.5-8 degrees eccentricity. What were the eccentricities of the expanding/contracting rings used to retinotopically map occipital cortical areas? How about the moving dots used to define MT+?

[Response] We appreciate this comment. We have added text in the Methods indicating that the expanding/contracting rings used in retinotopy ranged from 0.5-8.25 degrees of eccentricity (line 190). We have also specified that the dots in the MT+ localizer covered 0.5-10 degrees of eccentricity (line 211).

Was any attempt made to use a visual localizer to restrict the voxels in each visual field map to those that exhibited a visual response to the annulus used in the main experiment? If not, what might be the consequences of using ROIs that do not correspond to the visual field locations of the annulus? What if there are negative BOLD responses in cortical regions in the ROI that represent unstimulated visual field locations that are adjacent to the borders of the stimulus?

[Response] Thank you for this comment. We did not use a visual localizer to select voxels in our study. We have run additional analyses to assess the impact of voxel selection. We used the radial and polar angle maps obtained from the independent retinotopic mapping session to calculate each voxel's response field (RF) location, and included voxels that fell within three inclusion zones (zone 1: 2.5-8°; zone 2: 2.75-7.75°; zone 3: 3-7.5°), such that voxels falling outside the annulus were excluded. Because receptive fields in higher visual areas (e.g., IPS1) are large enough to cover the entire annulus and its surrounding area, we performed these analyses on V1, extrastriate areas (V2-V4), MT+, and V7. Below, we show the results of the correlation analysis with each inclusion zone. All areas still exhibited a significant difference between correct and incorrect trials (zone 1: all t(11) > 2.78, p=0.018; zone 2: all t(11) > 2.34, p=0.04; zone 3: all t(11) > 2.53, p=0.028). Therefore, these results show that voxels outside the annulus do not significantly impact our results.

Regarding negative BOLD, it is worth noting that negative BOLD responses also could carry information about the stimulus, such as contrast (Shmuel et al., 2002) and spatial separation (Bressler et al., 2007). Furthermore, attention produced similar effects in both positive and negative BOLD responses (Bressler et al., 2013). It seems to us that the functional significance of negative BOLD responses is unclear at this point and such responses could still be meaningful. Regardless of its status, the above re-analysis would have excluded voxels just outside the stimulus aperture which tend to show large negative BOLD responses. Hence, we feel our results are unlikely due to negative BOLD responses from these voxels.

It's unclear to me that an r-squared threshold combined with a cluster constraint for defining ROIs is equivalent to an independent visual localizer. This should be discussed by the authors.

[Response] We apologize for the confusion. The r2 threshold and cluster constraint were used for visualization purposes only (lines 333-335), not to define any ROIs.

5) It appears that separate two-way ANOVAs were conducted for each brain area in the analysis of univariate response amplitude. Shouldn't there be a correction for multiple comparisons in this case? There are at least 8 ROIs, and perhaps 11, depending on whether the extrastriate cortical areas were combined for this analysis. A Bonferroni correction would be incorrect, as the ROIs are not independent from one another, but a false discovery rate analysis seems appropriate. It appears that a correction for multiple comparisons (again, number of ROIs) should also be used for the fMRI correlation and classification analyses.

[Response] We appreciate this comment. While we have performed statistical tests in 8 brain areas, these are pre-defined ROIs. All occipital and parietal areas were defined from independent datasets and the two frontal areas were defined using orthogonal criteria from the main analyses. Importantly, these regions are also well-known functional areas of the brain that have been repeatedly found in many previous studies on visual attention (including our own). Indeed, we have set out to examine a priori how neural signals in these regions vary with task performance. Thus, our analyses are quite different from massive multiple post-hoc statistical tests such as that incurred by whole-brain voxel-wise analysis. We have added some emphasis in the Introduction to list the brain regions that have been found in previous studies and that are also expected to be our target regions for investigation (lines 54-56). Another strength of our study is that we have data from two very different methods (fMRI and TMS) that provided converging evidence. We recognize that the distinction between a priori and post-hoc analysis can be a little blurry. However, in this case, we believe our targeted analyses and converging evidence provide assurance against type I errors.

Minor points:

1) Abstract: “Human subjects discriminated a speedup...” The term “speedup” is not standard in the field, and many readers would not know that it refers to a change in speed of the moving dots. This should be clarified.

[Response] Thank you for pointing this out. We have changed “speedup” to “speed increment” in the Abstract (line 6).

2) Significance statement: “To cope with the metabolic limits of visual processing,...” Although neural activity is surely influenced by metabolic limits, this is not generally considered to be the fundamental basis of selective attention. I suggest changing “metabolic” to “computational”.

[Response] Thank you for the suggestion. We have changed “metabolic” to “computational” (line 21).

3) Materials and Methods: The term “best PEST” should be spelled out and explained.

[Response] We have added more information about best PEST in lines 122, and 124-127.

4) Materials and Methods: “The r-squared value indicated how much a voxel's time series was driven by the task.” This definition sounds more like a response amplitude measure. I think it would be more clear to explain this in terms of percent variance accounted for.

[Response] We agree. To make this clearer, we've changed our wording in line 252 from “explained” to “accounted for.”

5) Materials and Methods: “...separate clusters along the precentral sulcus: one near the superior frontal sulcus and another near the inferior frontal sulcus (see Figure 3A).” Figure 3A does not show two separate clusters. Later in the manuscript, it is clarified that the clusters are not separate on the group map but are “primarily distinct” in individual subjects. However, this text in the Materials and Methods section s confusing as currently written.

[Response] Thank you for raising this point. We have clarified the text in the Methods (lines 257-258).

6) In the TMS experiments, why was IPS1 targeted bilaterally but MT+ only in the right hemisphere?

[Response] We apologize for the confusion. All ROIs were targeted unilaterally in separate sessions. To emphasize this point, we've altered the description of the TMS experiments to explicitly state that ROIs were targeted unilaterally (lines 82-83, 349, 380, 521, and 530).

7) Materials and Methods: “Each area was topographically defined in individual subjects using the retinotopic mapping procedure.” This is true for IPS1 but not for MT+.

[Response] Thank you for pointing this out. We've changed that sentence to reflect that MT+ was defined using the motion localizer (lines 381-382).

8) Results: “Prior to TMS sessions, speedup magnitude thresholds for the attention and baseline tasks were obtained for each subject such that task difficulty was equated.” Does “task difficulty” refer to “hit rate” here? This should be clarified.

[Response] Thank you for pointing this out. We've changed “task difficulty” to “hit rate” (line 531).

References

Bressler D, Spotswood N, Whitney D (2007) Negative BOLD fMRI Response in the Visual Cortex Carries Precise Stimulus-Specific Information. PLoS ONE 2:e410.

Bressler DW, Fortenbaugh FC, Robertson LC, Silver MA (2013) Visual spatial attention enhances the amplitude of positive and negative fMRI responses to visual stimulation in an eccentricity-dependent manner. Vision Res 85:104--112.

Colman AM (2015) A dictionary of psychology. Oxford University Press, USA.

Shmuel A, Yacoub E, Pfeuffer J, Van de Moortele P-F, Adriany G, Hu X, Ugurbil K (2002) Sustained negative BOLD, blood flow and oxygen consumption response and its coupling to the positive response in the human brain. Neuron 36:1195--1210.
